# *TNFAIP8L1* and *FLT1* polymorphisms alter the susceptibility to cervical cancer amongst uyghur females in China

**DOI:** 10.1042/BSR20191155

**Published:** 2019-07-19

**Authors:** Lili Han, Sulaiya Husaiyin, Chunhua Ma, Lin Wang, Mayinuer Niyazi

**Affiliations:** Department of Gynecology, People’s Hospital of Xinjiang Uygur Autonomous Region, Urumqi, Xinjiang 830001, China

**Keywords:** cervical cancer risk, FLT1, single nucleotide polymorphisms, TNFAIP8L1, Uyghur females

## Abstract

*TNFAIP8L1* and *FLT1* play critical roles in the occurrence and development of tumors, but no in-depth studies have been carried out in cervical cancer. The present study aims to research the correlation between polymorphisms of these two genes and the risk of cervical cancer in the Uygur women. The study involved 342 cervical cancer patients and 498 healthy women. Five single nucleotide polymorphisms (SNPs) from the *TNFAIP8L1* gene and the *FLT1* gene were selected and genotyped. Odds ratio and 95% CIs were calculated by logistic regression analysis to evaluate the correlation between SNPs and cervical cancer risk. The alleles rs9917028-A (*P=*0.032), rs10426502-A (*P=*0.007), and rs1060555-G (*P=*0.026) of *TNFAIP8L1* were associated with a decreased risk of cervical cancer. In the multiple genetic models, these three SNPs were also associated with the risk of cervical cancer. The stratified analysis showed that *TNFAIP8L1*-rs10426502, -rs1060555, and *FLT1*-rs9513111 were associated with a decreased risk of cervical cancer amongst people older than 43 years. Moreover, the haplotypes AG (*P=*0.007) and GC (*P=*0.026) of linkage disequilibrium block rs10426502|rs1060555 in *TNFAIP8L1* were significantly associated with an increased risk of cervical cancer. Our results suggested that the relationships between *TNFAIP8L1* and *FLT1* polymorphisms and the risk of cervical cancer amongst Uyghur females.

## Introduction

Cervical cancer is the third most common cancer amongst women worldwide, with 527,624 new cases and 265,672 deaths in 2012 [1]. It is estimated that by 2020, there will be 609,270 new cases and 317,727 deaths worldwide [2]. While cancer-related mortality has decreased with the implementation of screening programs worldwide, incidence is on the rise in developing countries, where about 80% of cases occur [3]. China is a multiethnic country, the incidence of cervical cancer in all ethnic groups is different, and of which Xinjiang Uygur population incidence is the highest. In recent years, much attention has been paid to the study of cervical cancer in Uygur women [4]. However, the pathogenesis of cervical cancer was not fully understood. Etiological studies have shown that cervical cancer is the result of a combination of factors, including the high-risk human papillomavirus (HPV) infection, environmental, behavioral, and genetic factors [5,6]. In recent years, increasing evidence has emphasized the role of genetic factors in the pathogenesis of cervical cancer. Studies based on single nucleotide polymorphisms (SNPs) and genome-wide association studies have confirmed the relationship between genetic variations and the risk of cervical cancer in differen*P-*t populations [7–9].

Tumor necrosis factor (TNF)-α- induced protein 8 (TNFAIP8) is a recently identified protein family reported to have important roles in immunity, inflammation, and tumorigenesis. TNFAIP8L1 is a member of the TNFAIP8 family [10]. Past studies have shown a strong correlation between TNFAIP8 protein and the development of a lvariety of cancers, including gynecological cancers such as breast cancer, cervical cancer, ovarian cancer, and endometrial cancer [11]. In addition, the regulation of these proteins has been found to promote basic characteristics of cancer, such as tumor growth, proliferation, inhibition of apoptosis, and angiogenesis [12]. However, the exact function of *TNFAIP8L1* in cervical cancer is unknown. The *FLT1* gene encodes VEGFR1, a member of the vascular endothelial growth factor receptor (VEGFR) family. The VEGF system is crucial for angiogenesis, which is considered to be the key to the occurrence of malignant tumors [13,14]. A previous study showed that *VEGF* is an important regulator of endometrial tumor angiogenesis, and *FLT1* is a great marker of vascular proliferation [15,16]. At present, the function of *FLT1* in cervical cancer has not been deeply studied.

In this case-control study, we investigated the association between SNPs of *TNFAIP8L1* and *FLT1* genes and cervical cancer risk amongst Xinjiang Uygur females.

## Materials and methods

### Study population

A total of 342 patients were recruited from the People’s Hospital of Xinjiang Uygur Autonomous Region as preliminary samples. All patients were pathologically confirmed with cervical cancer by at least two pathologists. The International Federation of Gynecology and Obstetrics (FIGO) stage was also recorded for further analysis. Notably, patients who received systemic or topical treatment were excluded from our study. In addition, women eligible for age and ethnicity were recruited continuously from the same hospital health screening center as a control group. Finally, the study included 498 healthy individuals with no history of gynecologic disease or tumors. All participants were from Xinjiang Uygur ethnic group.

### DNA genotyping

The total DNA isolation was performed from the peripheral blood samples provided by the experimental subjects using the GoldMag DNA Purification Kit (GoldMag Co. Ltd, Xi’an City, China). The concentration and quality of the purified DNA were measured with Nanodrop 2000 UV spectrophotometer (Thermo Scientific, Waltham, MA, U.S.A.). Screening the polymorphisms with minor allele frequency (MAF) more than 5% in the 1000 genomes database (http://www.internationalgenome.org/) and the dbSNP database (https://www.ncbi.nlm.nih.gov/projects/SNP/). We eventually selected five SNPs including rs9917028, rs10426502, and rs1060555 of *TNFAIP8L1* and rs9513111 and rs677471 of *FLT1* for further genotype identification and risk association analysis. The Agena Bioscience Assay Design Suite software, version 2.0 (https://agenacx.com/online-tools/) was applied for MassARRAY assay design. The SNP genotype was identified by the MassARRAY Nanodispenser and MassARRAY iPLEX method (Agena Bioscience, San Diego, CA, U.S.A.) according to the manufacturer’s instructions. Data management and presentation were conducted by the Agena Bioscience TYPER software, version 4.0 [17–19].

### Statistical analysis

All the basic statistical analyses were carried out using SPSS 20.0 (SPSS, Chicago, IL, U.S.A.) and Microsoft Excel. Age distribution differences between cases and healthy controls were analyzed by the independent sample *t*-test. The departure from Hardy-Weinberg equilibrium (HWE) was determined by comparing the observed and expected heterozygosity on controls with the Chi-square test. Statistical significance was defined as *P*-value less than 0.05. All *P*-values were two tailed. Furthermore, the association study was performed in multiple inheritance models (co-dominant, dominant, recessive, and additive) using SNPstats software (http://bioinfo.iconcologia.net/SNPstats). Odds ratio (ORs) values and 95% CIs were calculated using an age-adjusted logistic regression to assess the risk of cervical cancer. Haploview 4.2 software (Cambridge, MA, U.S.A.) was used to determine the pairwise linkage disequilibrium (LD), using the standardized D′ and r^2^ values. The false-positive report probability (FPRP) was calculated to evaluate the significant results using the SAS software 9.4 (SAS Institute, Cary, N.C., U.S.A.). We set 0.2 as the FPRP threshold to detect an OR of 0.67/1.50 (protective/risk effect) associated with genotype and haplotype in the study. The FPRP value of less than 0.2 was considered a significant finding [20,21].

## Results

### Characteristics of the study subjects

In the present study, the age distribution was matched between the case group and the control group (*P*>0.05). A total of 342 cervical cancer patients and 498 healthy subjects were included, with the average age of 43.46 and 43.27, respectively (Table 1). Moreover, the frequency distribution of the cervical cancer patients regarding FIGO stage and HPV status were calculated and listed in Table 1.

**Table 1:** Characteristics of the study population

Variable	Cases	Controls	*P*-value
**Age (mean ± S.D.)**	43.46 ± 13.03	43.27 ± 11.78	0.829
≤43	166 (49%)	235 (53%)	
>43	176 (51%)	263 (47%)	
**Stage**			
I–II	132 (39%)		
III–IV	80 (23%)		
Absent	130 (38%)		
**HPV status**			
Negative	51 (15%)		
Positive	195 (57%)		
Absent	96 (28%)		
**Total**	342	498	

*P*-value obtained from independent sample *t*-test.

### Basic information for the candidate SNPs

Basic information and preliminary statistical results of the selected SNPs are showed in Table 2. HWE *P*-values were obtained with Chi-square tests and all SNPs (rs9917028, rs10426502, rs1060555, rs9513111, and rs677471) were in HWE (*P*>0.05). The values of the MAF were higher than 5%.

**Table 2:** Basic information regarding candidate SNPs

SNP	Chromosome	Position	Alleles	Gene	Role	MAF		HWE	OR	*P-*value
						Case	Control	*P-*value	(95% CI)	
rs9917028	Chr19	4640971	G/A	*TNFAIP8L1*	Intron	0.330	0.382	0.107	**0.80 (0.65-0.98)**	**0.032**
rs10426502	Chr19	4651257	G/A	*TNFAIP8L1*	Intron	0.037	0.067	0.154	**0.53 (0.33-0.84)**	**0.007**
rs1060555	Chr19	4652810	C/G	*TNFAIP8L1*	3′UTR	0.238	0.287	0.154	**0.78 (0.62-0.97)**	**0.026**
rs9513111	Chr13	28423426	C/T	*FLT1*	Intron	0.326	0.674	0.766	0.91 (0.74–1.12)	0.385
rs677471	Chr13	28489675	C/T	*FLT1*	Intron	0.355	0.645	0.160	1.08 (0.88–1.33)	0.448

*P-*values were calculated with Chi-square tests. *P<*0.05 indicates statistical significance.

### SNPs and cervical cancer risk

The differences in allele frequency between cases and controls were compared by χ^2^ test (Table 2). The allele with lower frequency of each SNP was regarded as a risk factor. The results indicated that alleles rs9917028-A (*P=*0.032), rs10426502-A (*P=*0.007), and rs1060555-G (*P=*0.026) of *TNFAIP8L1* were risk alleles for cervical cancer amongst Xinjiang Uygur female.

As showed in Table 3, significant associations were existed between *TNFAIP8L1* rs9917028, and decreased risk of cervical cancer in allele model, co-dominant model, and additive model (allele model: OR = 0.80, 95% CI: 0.65–0.98, *P=*0.032; co-dominant model: OR = 0.64, 95% CI: 0.42–0.99, *P=*0.044; additive model: OR = 0.81, 95% CI: 0.66–0.99, *P=*0.039). *TNFAIP8L1* rs10426502 and rs1060555 were decreased risk of cervical cancer in allele model, co-dominant model, dominant model, and additive model (rs10426502: allele model: OR = 0.53, 95% CI: 0.33–0.84, *P=*0.007; co-dominant model: OR = 0.47, 95% CI: 0.28–0.76, *P=*0.003; dominant model: OR = 0.49, 95% CI: 0.30–0.79, *P=*0.004; additive model: OR = 0.52, 95% CI: 0.32–0.83, *P=*0.007. rs1060555: allele model: OR = 0.78, 95% CI: 0.62–0.97, *P=*0.026; co-dominant model: OR = 0.70, 95% CI: 0.52–0.93, *P=*0.015; dominant model: OR = 0.70, 95% CI: 0.53–0.93, *P=*0.012; additive model: OR = 0.77, 95% CI: 0.61–0.97, *P=*0.024).

**Table 3:** The relationship between *TNFAIP8L1* gene polymorphism and cervical cancer

SNP	Model	Genotype	Case	Control	Adjusted by age	
					OR (95% CI)	*P-*value
rs9917028	Co-dominant	GG	157 (46%)	199 (40%)	1	
		GA	144 (42%)	218 (44%)	0.84 (0.62–1.13)	0.241
		AA	41 (12%)	81 (16%)	**0.64 (0.42–0.99)**	**0.044**
	Dominant	GG	157 (46%)	199 (40%)	1	
		GA–AA	185 (54%)	299 (60%)	0.78 (0.59–1.04)	0.088
	Recessive	GG-GA	301 (88%)	417 (84%)	1	
		AA	41 (12%)	81 (16%)	0.70 (0.47–1.05)	0.087
	Additive	—	—	—	**0.81 (0.66–0.99)**	**0.039**
rs10426502	Co-dominant	GG	317 (93%)	431 (83%)	1	
		GA	23 (6%)	67 (17%)	**0.47 (0.28–0.76)**	**0.003**
		AA	1 (1%)	0 (0%)	—	0.999
	Dominant	GG	317 (93%)	431 (83%)	1	
		GA-AA	24 (7%)	67 (17%)	**0.49 (0.30–0.79)**	**0.004**
	Recessive	GG-GA	341 (91.90%)	498 (100%)	1	
		AA	1 (1%)	0 (0%)	—	0.999
	Additive	—	—	—	**0.52 (0.32–0.83)**	**0.007**
rs1060555	Co-dominant	CC	199 (58%)	246 (49%)	1	
		CG	123 (36%)	218 (44%)	**0.70 (0.52–0.93)**	**0.015**
		GG	20 (6%)	34 (7%)	0.73 (0.41–1.30)	0.285
	Dominant	CC	199 (58%)	246 (49%)	1	
		CG-GG	143 (42%)	252 (57%)	**0.70 (0.53–0.93)**	**0.012**
	Recessive	CC-CG	322 (94%)	464 (93%)	1	
		GG	20 (6%)	34 (7%)	0.85 (0.48–1.50)	0.571
	Additive	—	—	—	**0.77 (0.61–0.97)**	**0.024**

*P-*values were calculated with wald-test. *P<*0.05 indicates statistical significance.

Based on age stratification, we tried to determine the effect of these variants on the risk of cervical cancer, as showed in Table 4. In accordance with our statistically significant findings of the allele model, the minor allele of rs10426502, and rs1060555 in *TNFAIP8L1* played roles in reducing the risk of cervical cancer in Uygur women over 43 years old (rs10426502: OR = 0.44, 95% CI: 0.22–0.88, *P=*0.017; rs1060555: OR = 0.72, 95% CI: 0.53–0.97, *P=*0.033). Amongst Uygur women under 43 years of age, the rs9917028-‘AA’ genotype of *TNFAIP8L1* reduced the risk of cervical cancer in genotype model and additive model (genotype model: OR = 0.45, 95% CI: 0.22–0.91, *P=*0.026; additive model: OR = 0.47, 95% CI: 0.24–0.91, *P=*0.025). Amongst Uygur women over 43 years of age, individuals carrying the heterozygous genotype rs1060555-‘CG’ in *TNFAIP8L1* (OR = 0.40, 95% CI: 0.17–0.92, *P=*0.017) were less likely to suffer from cervical cancer when compared with the homozygous ‘CC’. In addition, rs10426502 and rs1060555 of *TNFAIP8L1* were significantly associated with reduced risk of cervical cancer in the dominant and additive models, as well as rs9513111 of *FLT1* was significantly associated with reduced risk of cervical cancer in the recessive model (*P*<0.05).

**Table 4:** Relationships between *TNFAIP8L1* and *FLT1* polymorphism and cervical cancer risk according to the stratification by age

SNP	Model	Genotype	≤43		>43	
Gene			OR (95% CI)	*P-*value	OR (95% CI)	*P-*value
rs9917028	Allele	G	1		1	
*TNFAIP8L1*		A	0.75 (0.56–1.02)	0.064	0.84 (0.64–1.12)	0.238
	Genotype	GG	1		1	
		GA	0.94 (0.61–1.43)	0.766	0.75 (0.50–1.15)	0.187
		AA	**0.45 (0.22–0.91)**	**0.026**	0.80 (0.46–1.40)	0.434
	Dominant	GG	1		1	
		GA-AA	0.81 (0.54–1.21)	0.305	0.77 (0.52–1.13)	0.180
	Recessive	GG-GA	1		1	
		AA	**0.47 (0.24–0.91)**	**0.025**	0.92 (0.55–1.55)	0.757
	Additive	—	0.75 (0.56–1.02)	0.065	0.86 (0.66–1.13)	0.281
rs10426502	Allele	G	1		1	
*TNFAIP8L1*		A	0.62 (0.33–1.19)	0.149	**0.44 (0.22–0.88)**	**0.017**
	Genotype	GG	1		1	
		GA	0.53 (0.26–1.07)	0.077	—	—
		AA	—	0.999	—	—
	Dominant	GG	1		1	
		GA-AA	0.57 (0.29–1.14)	0.112	**0.42 (0.21–0.86)**	**0.017**
	Recessive	GG-GA	1		1	
		AA	—	0.999	—	—
	Additive	—	0.63 (0.33–1.22)	0.173	**0.42 (0.21–0.86)**	**0.017**
rs1060555	Allele	C	1		1	
*TNFAIP8L1*		G	0.86 (0.62–1.19)	0.355	**0.72 (0.53–0.97)**	**0.033**
	Genotype	CC	1		1	
		CG	0.94 (0.62–1.42)	0.760	**0.54 (0.36–0.82)**	**0.003**
		GG	0.59 (0.22–1.60)	0.303	0.79 (0.38–1.64)	0.527
	Dominant	CC	1		1	
		CG-GG	0.89 (0.60–1.34)	0.583	**0.58 (0.39–0.85)**	**0.005**
	Recessive	CC-CG	1		1	
		GG	0.61 (0.23–1.62)	0.321	1.03 (0.50–2.10)	0.935
	Additive	—	0.87 (0.61–1.22)	0.406	**0.71 (0.52–0.97)**	**0.030**
rs9513111	Allele	T	1		1	
*FLT1*		C	1.05 (0.78–1.41)	0.773	0.81 (0.61–1.08)	0.144
	Genotype	TT	1		1	
		TC	0.89 (0.58–1.36)	0.581	1.02 (0.68–1.54)	0.913
		CC	1.38 (0.69–2.76)	0.359	0.55 (0.29–1.04)	0.067
	Dominant	TT	1		1	
		TC-CC	0.96 (0.64–1.44)	0.852	0.89 (0.61–1.31)	0.561
	Recessive	TT-TC	1		1	
		CC	1.47 (0.76–2.83)	0.250	**0.54 (0.29–0.99)**	**0.049**
	Additive	—	1.06 (0.78–1.45)	0.695	0.82 (0.62–1.09)	0.166

*P-*values were calculated with wald-test. *P*<0.05 indicates statistical significance.

### Haplotype analyses and false-positive report

**Figure 1:**
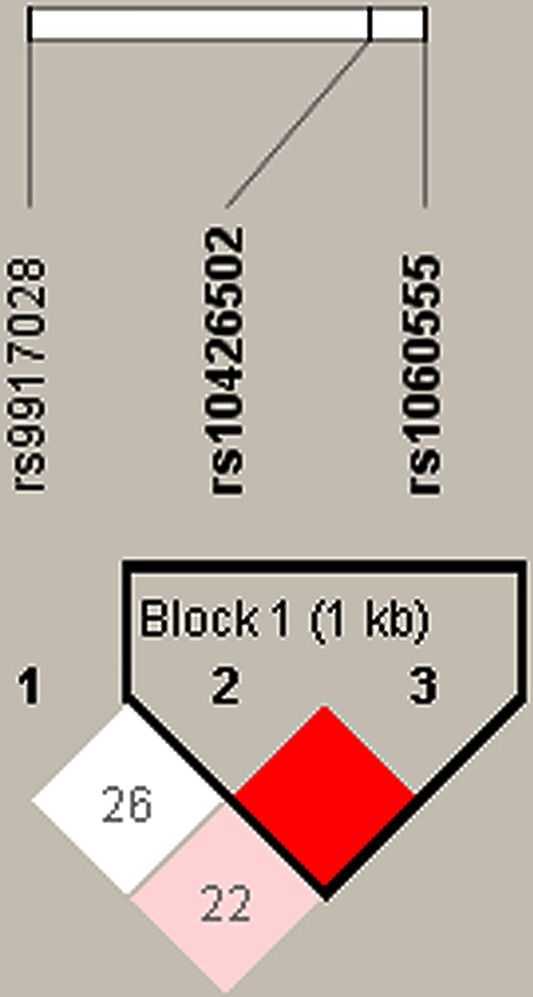
LD block construction Block rs10426502-rs1060555 was detected in *TNFAIP8L1*. (D′ = 1.0, r^2^ = 0.159).

Furthermore, haplotype analyses were performed and a LD block was found in the *TNFAIP8L1* gene, formed by rs10426502-rs1060555 (Figure 1). The haplotypes AG (OR = 1.94, 95% CI: 0.20–3.13, *P=*0.007) and GC (OR = 1.30, 95% CI: 1.03–1.63, *P=*0.026) were significantly associated with an increased risk for cervical cancer in Uyghur population (Table 5).

**Table 5:** Haplotype frequencies of *TNFAIP8L1* SNPs and the association with cervical cancer risk

Gene	Haplotype	Haplotype frequency		OR (95% CI)	*P-*value
		Case	Control		
	rs10426502|rs1060555				
*TNFAIP8L1*	_AG_	0.9633	0.933	**1.94 (1.20–3.13)**	**0.007**
	_GG_	0.7977	0.780	1.11 (0.87–1.42)	0.386
	_GC_	0.761	0.713	**1.30 (1.03–1.63)**	**0.026**

*P-*values were calculated with Pearson’s Chi-square tests. *P<*0.05 indicates statistical significance.

FPRP values at different prior probability levels were calculated to evaluate significance results (Supplementary Table S1). For a prior probability of 0.1, the FPRP values were less than 0.2 for the associations of rs10426502, rs1060555 alleles, and genotypes with decreased risk of cervical cancer (rs10426502: allele-A: 0.094; GA: 0.045; GA-AA: 0.062. rs1060555: allele-G: 0.187 CG: 0.112; CG-GG: 0.112). And the FPRP values were also less than 0.2 for the associations of haplotypes with increased risk of cervical cancer (rs10426502|rs1060555: AG: 0.098; GC: 0.172). In contrast, we observed greater FPRP values for the significant associations between rs9917028 and cervical cancer risk, suggesting some possible bias in the findings due to limited sample size, which need further validation in larger sample.

## Discussion

In the present study, selected SNPs in *TNFAIP8L1* and *FLT1* and their association with cervical cancer risk were investigated for the first time. Three *TNFAIP8L1* variants and one variant of *FLT1* were associated with reduced cervical cancer susceptibility amongst females from Xinjiang Uyghur Autonomous Region of China.

*TNFAIP8L1* plays an important role in malignant tumor. Immunohistochemistry and western blot analysis showed that TNFAIP8L1-specific antibodies were expressed in both male and female reproductive organs and germ cell tissues of mice. In addition, elevated *TNFAIP8L1* mRNA levels were detected in human gynecological cancer cells, including cervical and ovarian cancer cells [22]. As a regulator of cell death, *TNFAIP8L1* plays an indispensable role in tumor cell necrosis. Studies have shown that *TNFAIP8L1* can induce hepatocellular carcinoma cell apoptosis by interacting with Rac1 [23,24]. In support of this view, *TNFAIP8L1* expression was down-regulated in hepatocellular carcinoma tissues compared with adjacent normal tissues. In addition, *TNFAIP8L1* was found to significantly reduce tumor burden *in vivo* and cell proliferation *in vitro*. Similar to hepatocellular carcinoma, down-regulation of *TNFAIP8L1* has also been observed in lung cancer [12]. Our study showed that these three candidate SNPs (rs9917028, rs10426502, and rs1060555) were risk factors for cervical cancer amongst Uygur women in Xinjiang. We hypothesized that these three SNPs might affect the expression and function of *TNFAIP8L1* in the development of cervical cancer.

The development and metastasis of primary tumors require angiogenesis, growth, movement, and detachment. Therefore, angiogenesis, the formation of blood vessels, has been identified as the key to the occurrence and development of malignant tumors. VEGF is considered as an important regulator of angiogenesis [15,25]. VEGF receptor 1 (FLT1) is one of the targets of VEGF, which can regulate the growth and migration of endothelial cells and regulate angiogenesis. Recent studies have shown that FLT1 present and functional in different human cancer cells, and VEGF activation of FLT1 can be involved in the process of tumor progression [26–28]. *FLT1* has been shown to be a marker of angiogenesis in endometrial cancer, but its function as a predictor has not been determined. Daniel et al. believed that the *FLT1*-snp allele might be an important risk factor for angiogenesis-related disease [15]. Currently, VEGFA and its receptor-FLT1, as pro-angiogenic growth factor, are related to the promotion of angiogenesis, vascular permeability, cell migration and gene expression, and have become the targets of anticancer treatment [29]. Our findings suggest that *FLT1* rs9513111 may serve as a clinical predictor in Uygur women over 43 years of age. Our experimental results should be further verified in a larger sample size

There are still some limitations in the present study. First, this work detected the effect of SNP of two genes on cervical cancer susceptibility, and the specific molecular mechanism needs to be further explored. Second, lack of other clinical information. Therefore, further studies with a larger sample size and functional experiments are required.

Our study validated the relationship between *TNFAIP8L1* and *FLT1* variations and the risk of cervical cancer in Uygur women. These SNPs (rs9917028, rs10426502, rs1060555, and rs9513111) are expected to be new targets for early diagnosis and prevention of cervical cancer in Uygur women.

## Supporting information

**Supplementary Table S1:** False-Positive Report Probability Values for associations between the risk of cervical cancer and the frequency of genotypes and haplotypes

## Abbreviations

FIGO: International Federation of Gynecology and Obstetrics
FPRP: false-positive report probability
HPV: high-risk human papillomavirus
HWE: Hardy-Weinberg equilibrium
LD: linkage disequilibrium
MAF: minor allele frequency
TNF: tumor necrosis factor
VEGFR: vascular endothelial growth factor receptor

## References

[1] Bray F., Ferlay J., Soerjomataram I., Siegel R.L., Torre L.A. and Jemal A. (2018) Global cancer statistics 2018: GLOBOCAN estimates of incidence and mortality worldwide for 36 cancers in 185 countries. CA Cancer J. Clin. 68, 394–424 10.3322/caac.21492 30207593

[2] Vargastorres S.L., Portari E.A., Silva A.L., Klumb E.M., Hc D.R.G., Mj D.C. (2016) Roles of CDKN1A gene polymorphisms (rs1801270 and rs1059234) in the development of cervical neoplasia. Tumour Biol. 37, 10469–10478 10.1007/s13277-016-4850-3 26846214

[3] Waggoner S.E. (2003) Cervical cancer. Lancet 361, 2217–2225 10.1016/S0140-6736(03)13778-6 12842378

[4] Abulizi G., Abulimiti T., Li H., Abuduxikuer G., Mijiti P., Zhang S. (2018) Knowledge of cervical cancer and Pap smear amongst Uyghur women from Xinjiang, China. BMC Women’s Health 18, 21 10.1186/s12905-018-0512-5 29343254 PMC5773149

[5] De Freitas A.C., Gurgel A.P.A.D., Chagas B.S., Coimbra E.C. and Amaral C.M.M.D. (2012) Susceptibility to cervical cancer: an overview. Gynecol. Oncol. 126, 304–311 10.1016/j.ygyno.2012.03.047 22484226

[6] Wang J., Chai Y., Wang T., Liu J., Dai P. and Liu Z. (2015) Genetic alterations of PIK3CA and tumor response in patients with locally advanced cervical squamous cell carcinoma treated with cisplatin-based concurrent chemoradiotherapy. Exp. Mol. Pathol. 98, 407–410 10.1016/j.yexmp.2015.03.014 25773678

[7] Kuguyo O., Tsikai N., Thomford N.E., Magwali T., Madziyire M.G., Nhachi C.F.B. (2018) Genetic susceptibility for cervical cancer in african populations: what are the host genetic drivers? OMICS 22, 468–483 10.1089/omi.2018.0075 30004844

[8] Miura K., Mishima H., Kinoshita A., Hayashida C., Abe S., Tokunaga K. (2014) Genome-wide association study of HPV-associated cervical cancer in Japanese women. J. Med. Virol. 86, 1153–1158 10.1002/jmv.23943 24700089

[9] Leo P.J., Madeleine M.M., Wang S. and Schwartz S.M. (2017) Defining the genetic susceptibility to cervical neoplasia-A genome-wide association study. PLoS Genet. 13, e1006866 10.1371/journal.pgen.1006866 28806749 PMC5570502

[10] Goldsmith J.R. and Chen Y.H. (2017) Regulation of inflammation and tumorigenesis by the TIPE family of phospholipid transfer proteins. Cell Mol. Immunol 14, 482–487 10.1038/cmi.2017.4 28287114 PMC5518821

[11] Lou Y. and Liu S. (2011) The TIPE (TNFAIP8) family in inflammation, immunity, and cancer. Mol. Immunol. 49, 4–7 10.1016/j.molimm.2011.08.006 21924498

[12] Padmavathi G., Banik K., Monisha J., Bordoloi D., Shabnam B., Arfuso F. (2018) Novel tumor necrosis factor-α induced protein eight (TNFAIP8/TIPE) family: Functions and downstream targets involved in cancer progression. Cancer Lett. 432, 260–271 10.1016/j.canlet.2018.06.017 29920292

[13] Menendez D., Krysiak O., Inga A., Krysiak B., Resnick M.A. and Schonfelder G. (2006) A SNP in the flt-1 promoter integrates the VEGF system into the p53 transcriptional network. Proc. Natl Acad. Sci. U.S.A. 103, 1406–1411 10.1073/pnas.0508103103 16432214 PMC1360546

[14] Levi J.A. (1987) Cancer: principles and practice of oncology. Patho 19, 109 10.1016/S0031-3025(16)39749-5

[15] Fine B.A., Valente P.T., Feinstein G.I. and Dey T. (2000) VEGF, flt-1, and KDR/flk-1 as prognostic indicators in endometrial carcinoma. Gynecol. Oncol. 76, 33–39 10.1006/gyno.1999.5658 10620438

[16] Glubb D.M., Parebrunet L., Jantuslewintre E., Jiang C., Crona D.J., Etheridge A.S. (2015) Functional FLT1 genetic variation is a prognostic factor for recurrence in stage I–III non–small-cell lung cancer. J. Thorac. Oncol. 10, 1067–1075 10.1097/JTO.0000000000000549 26134224 PMC4494119

[17] Xia P., Li B., Geng T., Deng Z., Dang C., Chang D. (2015) FGFR2 gene polymorphisms are associated with breast cancer risk in the Han Chinese population. Am. J. Cancer Res. 5, 1854–1861 26175953 PMC4497451

[18] Ren H.T., Li Y.M., Wang X.J., Kang H.F., Jin T.B., Ma X.B. (2016) PD-1 rs2227982 polymorphism is associated with the decreased risk of breast cancer in Northwest Chinese women: a hospital-based observational study. Medicine 95, e3760 10.1097/MD.0000000000003760 27227944 PMC4902368

[19] Zhou L., He N., Feng T., Geng T., Jin T. and Chen C. (2015) Association of five single nucleotide polymorphisms at 6q25.1 with breast cancer risk in northwestern China. Am. J. Cancer Res. 5, 2467–2475 26396922 PMC4568782

[20] Wacholder S., Chanock S., Garcia-Closas M., Ghormli L. El and Rothman N. (2004) Assessing the probability that a positive report is false: an approach for molecular epidemiology studies. J. Natl Cancer Inst. 96, 434–442 10.1093/jnci/djh075 15026468 PMC7713993

[21] He J., Wang M.Y., Qiu L.X., Zhu M.L., Shi T.Y., Zhou X.Y. (2013) Genetic variations of mTORC1 genes and risk of gastric cancer in an Eastern Chinese population. Mol. Carcinog. 52, 70–79 10.1002/mc.22013 23423739

[22] Cui J., Zhang G., Hao C., Wang Y., Lou Y., Zhang W. (2011) The expression of TIPE1 in murine tissues and human cell lines. Mol. Immunol. 48, 1548–1555 10.1016/j.molimm.2011.04.023 21600655

[23] Zhang Z., Liang X., Gao L., Ma H., Liu X., Pan Y. (2015) TIPE1 induces apoptosis by negatively regulating Rac1 activation in hepatocellular carcinoma cells. Oncogene 34, 2566–2574 10.1038/onc.2014.208 25043299

[24] Wu X., Ma Y., Cheng J., Li X., Zheng H., Jiang L. (2017) TIPE1 function as a prognosis predictor and negative regulator of lung cancer. Oncotarget 8, 78496–78506 29108244 10.18632/oncotarget.19655 PMC5667977

[25] Punt S., Houwing-Duistermaat J.J., Schulkens I.A., Thijssen V.L., Osse E.M., de Kroon C.D. (2015) Correlations between immune response and vascularization qRT-PCR gene expression clusters in squamous cervical cancer. Mol. Cancer 14, 71 10.1186/s12943-015-0350-0 25889974 PMC4392729

[26] Hiratsuka S., Nakamura K., Iwai S., Murakami M., Itoh T., Kijima H. (2002) MMP9 induction by vascular endothelial growth factor receptor-1 is involved in lung-specific metastasis. Cancer Cell 2, 289–300 10.1016/S1535-6108(02)00153-8 12398893

[27] Fan F., Wey J.S., McCarty M.F., Belcheva A., Liu W., Bauer T.W. (2005) Expression and function of vascular endothelial growth factor receptor-1 on human colorectal cancer cells. Oncogene 24, 2647–2653 10.1038/sj.onc.1208246 15735759

[28] Wey J.S., Fan F., Gray M.J., Bauer T.W., McCarty M.F., Somcio R. (2005) Vascular endothelial growth factor receptor-1 promotes migration and invasion in pancreatic carcinoma cell lines. Cancer 104, 427–438 10.1002/cncr.21145 15952180

[29] Slattery M.L., Lundgreen A. and Wolff R.K. (2014) VEGFA, FLT1, KDR and colorectal cancer: assessment of disease risk, tumor molecular phenotype, and survival. Mol. Carcinog. 53, E140–50 10.1002/mc.22058 23794399

