# Supplementary material for: TNFAIP8L1 and FLT1 polymorphisms alter the susceptibility to cervical cancer amongst uyghur females in China
Source: Biosci Rep. 2019 Jul 19;39(7):BSR20191155. doi: 10.1042/BSR20191155 (PMC6639457; doi:10.1042/BSR20191155)
Supplement: Supplementary file 1 [file bsr20191155_Supp1.pdf]

Supplementary table 1 False-Positive Report Probability Values for associations between the risk of cervical cancer and the frequency of genotypes and haplotypes

Genotype/haplotype	OR (95% CI)	*p*-value	Statistical power	Prior probability				
				0.25	0.1	0.01	0.001	0.0001
**rs9917028**								
A vs. G	0.80(0.65 - 0.98)	0.032	0.961	**0.085**	0.219	0.755	0.969	0.997
AA vs. GG	0.64 (0.42 - 0.99)	0.044	0.866	**0.135**	0.318	0.837	0.981	0.998
**rs10426502**								
A vs. G	0.53(0.33 - 0.84)	0.007	0.598	**0.033**	**0.094**	0.533	0.920	0.991
GA vs. GG	0.47 (0.28 - 0.76)	0.003	0.400	**0.015**	**0.045**	0.339	0.838	0.981
GA-AA vs. GG	0.49 (0.30 - 0.79)	0.004	0.467	**0.021**	**0.062**	0.420	0.880	0.987
**rs1060555**								
G vs. C	0.78(0.62 - 0.97)	0.026	0.921	**0.071**	**0.187**	0.716	0.962	0.996
CG vs. CC	0.70 (0.52 - 0.93)	0.015	0.632	**0.040**	**0.112**	0.581	0.933	0.993
CG-GG vs. CC	0.70 (0.53 - 0.93)	0.012	0.632	**0.040**	**0.112**	0.581	0.933	0.993
**Haplotype**: **rs10426502|rs1060555**								
AG vs. GG	1.94 (1.20 - 3.13)	0.007	0.550	**0.035**	**0.098**	0.544	0.923	0.992
GC vs. GG	1.30 (1.03 - 1.63)	0.026	0.892	**0.065**	**0.172**	0.695	0.958	0.996

OR: odds ratio, CI: confidence interval.

p values were calculated with wald-test. $p < 0.05$ indicates statistical significance.

Statistical power was calculated using the number of observations in the subgroup and the OR and *p*-values in this table
